# Beware of your “oocyte specific” Cre line: somatic cell Cre expression in several *Zp3–Cre* lines and the *Gdf9–iCre* transgenic line^†^

**DOI:** 10.1093/biolre/ioag093

**Published:** 2026-04-30

**Authors:** Paula Stein, Chihiro Emori, Elizabeth Padilla-Banks, Lenka Radonova, Artiom Gruzdev, Masahito Ikawa, Carmen J Williams

**Affiliations:** Reproductive and Developmental Biology Laboratory, National Institute of Environmental Health Sciences, National Institutes of Health, Durham, North Carolina, United States; Research Institute for Microbial Diseases, The University of Osaka, Osaka, Japan; Reproductive and Developmental Biology Laboratory, National Institute of Environmental Health Sciences, National Institutes of Health, Durham, North Carolina, United States; Reproductive and Developmental Biology Laboratory, National Institute of Environmental Health Sciences, National Institutes of Health, Durham, North Carolina, United States; Gene Editing and Mouse Model Core, National Institute of Environmental Health Sciences, National Institutes of Health, Durham, North Carolina, United States; Research Institute for Microbial Diseases, The University of Osaka, Osaka, Japan; Reproductive and Developmental Biology Laboratory, National Institute of Environmental Health Sciences, National Institutes of Health, Durham, North Carolina, United States

**Keywords:** oocyte, Zp3–Cre, Gdf9–iCre, conditional knockout, transgenic

## Abstract

Several zona pellucida 3 (*Zp3*)-Cre driver mouse lines are used to enable conditional loss-of-function studies in oocytes. The Knowles *Zp3-Cre* line, currently maintained live at the Jackson Laboratory, is the most widely published. We recently found that the transgene expressed in the Knowles line contains a truncated metallothionein 1 (*Mt1*) sequence that is expressed at high levels in oocytes from transgenic mice. This finding led us to search for an alternative *Zp3-Cre* line that did not express an exogenous *Mt1* transcript. We tested a second transgenic *Zp3-Cre* line and then created our own transgenic *Zp3-Cre* line, neither of which was oocyte-specific as documented by crossing to the tdTomato reporter line. Similar testing confirmed the exquisite oocyte specificity of the Knowles *Zp3-Cre* line. An alternative to *Zp3-Cre* is to use the growth differentiation factor 9 (*Gdf9*)*-iCre* line, which is reported to be oocyte-specific and expressed beginning at the primordial follicle stage. This line similarly showed high somatic tissue Cre expression. Reasoning that a knock-in approach would ensure oocyte specificity, we used a CRISPR/Cas9 approach to insert Cre into the endogenous *Zp3-Cre* locus. However, crosses of this knock-in line with tdTomato females revealed high somatic tissue expression. Both the *Zp3-Cre* knock-in and *Gdf9-iCre* alleles, when paternally inherited, induced Cre expression by the blastocyst stage, explaining the broad tissue distribution. We conclude that the Knowles *Zp3-Cre* transgenic line remains the best model for generating oocyte-specific expression, though investigators should be aware of the spurious *Mt1* expression from the transgene.

## Teaser

Many “oocyte-specific” Cre drivers are not oocyte specific.

## Graphical Abstract

**Graphical Abstract:**
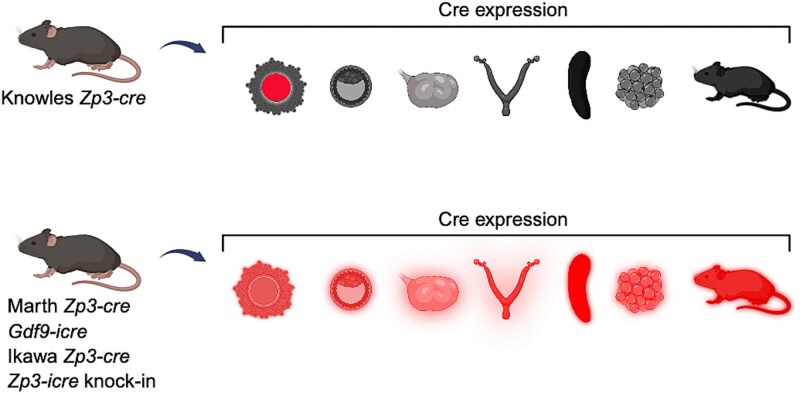

## Introduction

For researchers studying oocyte biology, mouse zona pellucida 3 (*Zp3*)-*Cre* transgenic lines provide an effective way to delete floxed sequences in growing oocytes within ovarian follicles. Several *Zp3*–*Cre* lines have been developed and characterized by different investigators. The first *Zp3*–*Cre* line reported was generated by Gail Martin’s group and published in 1997 [1]. This line was described as having female germ line-specific Cre expression as demonstrated using a polymerase chain reaction (PCR)-based assay, but the authors also reported some Cre expression in several somatic tissues (brain, ear, and tail). Further, females from the line were subfertile when homozygous, a problematic finding for studies of reproduction. A second mouse line made using the same construct had widespread somatic Cre expression [1], an observation that presaged the difficulties we were soon to encounter in our search for a truly oocyte-specific Cre mouse line.

A second *Zp3*–*Cre* line was published by Jamey Marth’s group in 2000 [2]. This line was generated for the purpose of excising a floxed sequence from the O-GlcNAc transferase gene locus on the X chromosome. A conditional knockout approach was necessary given the apparent cell lethality of the full deletion of *Ogt* in mouse embryonic stem (ES) cells and subsequent demonstration of embryonic lethality in vivo. How specificity of the *Zp3*–*Cre* line was tested was not described in the manuscript. However, the authors noted in the Materials and Methods section that “Cre recombination was found to be restricted to oocytes in female mice (data not shown)” [2].

Around the same time, *Zp3*–*Cre* transgenic mouse lines were developed by Barbara Knowles’ group using a *Zp3* promoter sequence similar to the one used in the Martin *Zp3*–*Cre* mouse line [3, 4]. These lines were tested for oocyte specificity by crossing to B6,129-Gtrosa^tm1Sor^ Cre reporter males [5]. β-galactosidase staining as evidence of Cre activity was observed only in growing oocytes of F1 hybrid females. Additional testing by PCR for Cre-mediated excision of a floxed β-catenin allele across numerous somatic tissues, in which deletion was not detected, confirmed the oocyte specificity [3]. One of these lines is maintained as a live colony at The Jackson Laboratory (JAX strain # 003651) and has been used widely to generate oocyte-specific conditional knockout mice.

When using the Knowles *Zp3*–*Cre* line ourselves, we became aware that the *Zp3*–*Cre* transgene includes most of the metallothionine 1 (*Mt1*) gene locus rather than just a poly(A) signal. The included sequence starts within *Mt1* intron 1 and has an in-frame ATG near the 5′ end of the transcript [6]. Very high expression of this alternative *Mt1* transcript in oocytes carrying the *Zp3*–*Cre* transgene led to our concern that an N-terminally modified MT1 protein could be expressed in conditional knockout oocytes and might impact their phenotypes. With this in mind, we wanted to find an oocyte-specific *Zp3*–*Cre* transgenic line that had an alternate poly(A) signal for use in our studies.

## Methods

### Animals and treatments

Knowles *Zp3*–*Cre*, Marth *Zp3*–*Cre*, *Gdf9*–*iCre*, and tdTomato transgenic mice were obtained from The Jackson Laboratory (strains # 003651, # 006888, # 011062, and # 007914, respectively; see Table 1). For oocyte collection, adult tdTomato females were primed with 7.5 IU equine chorionic gonadotropin (eCG; Lee Biosolutions), and cumulus–oocyte complexes (COCs) collected from the ovary as previously described [8]. For 1-cell embryo collection, tdTomato females were superovulated by intraperitoneal injection of 7.5 IU eCG, followed 48 h later by 7.5 IU human chorionic gonadotropin (hCG; Millipore-Sigma), and mating to males of the various mouse lines indicated in the text. For metaphase II egg collection, the same procedure was employed, but females were not mated after hCG injection. Eggs and 1-cell embryos were released from the oviductal ampulla at 14–16 h, or 20 h following hCG injection, respectively. Cumulus cells were removed by a brief incubation in 0.3% hyaluronidase, and eggs were washed and placed in a fresh drop of phosphate-buffered saline (PBS) for imaging. One-cell embryos were cultured to the blastocyst stage in Potassium Simplex Optimized Medium (KSOM, Millipore-Sigma) covered with mineral oil in a humidified atmosphere of 5% CO_2_ and 5% O_2_ at 37°C. Somatic tissues were isolated at the same time as oocytes or eggs, and small pieces placed in PBS for imaging. All animal procedures complied with National Institutes of Health animal care guidelines under an approved Animal Care and Use Committee protocol (RDBL07-37).

**Table 1:** Mouse lines discussed in this paper.

Full name	Our shorthand name	Promoter description	Additional sequence features	Source/ID	Comments
C57BL/6 J-Tg(*Zp3*–*Cre*)93Knw/J	Knowles *Zp3*–*Cre*	~6.5 kB promoter sequence (now available from Addgene; plasmid #14634)	Mt1 poly(A) signal	Jax strain #003651 RRID:IMSR_JAX:003651	[3]
B6.Cg-Tg(*Zp3*–*Cre*)1Gwh/J	Marth *Zp3*–*Cre*	6-kb SalI-PvuII fragment of *Zp3* promoter	human growth hormone splicing and polyadenylation signals	Jax strain #:006888 RRID:IMSR_JAX:006888	[2] Stated it was oocyte specific (data not shown)
C57BL/6 N-Tg(*Zp3*–*Cre*)1Osb	Ikawa *Zp3*–*Cre*	~6.5 kB promoter sequence (now available from Addgene; Plasmid #14634)	Bovine growth hormone poly(A) signal	RBRC #: 11257	(Masa Ikawa) This paper
B6.Cg-Zp3 < tm1(Cre)Cjwi>	*Zp3*–*Cre* knock-in	Endogenous *Zp3* promoter	Minimal SV40 poly(A) signal		(NIEHS Core) This paper
STOCK Tg(*Gdf9*–*iCre*)5092Coo/J	*Gdf9*–*iCre*	*Gdf9* promoter fragment (−3277 to +48)	simian virus 40 (SV40) large T nuclear localization signal and SV40 poly(A) signal	Jax strain #:011062 RRID:IMSR_JAX:011062	[11] Tested specificity with beta-galactosidase reporter; no signal in multiple somatic tissues
FVB/N-Tg(*Zp3*–*Cre*)3Mrt/J			Human beta-actin	Jax strain #:003377 RRID:IMSR_JAX:003377	[1] reports widespread somatic Cre expression in second mouse line made using same construct and some Cre expression in brain, ear, and tail in the published line.
B6.Cg-Gt(ROSA)26Sortm14(CAG-tdTomato)Hze/J	Ai14			JAX strain #007914 RRID:IMSR_JAX:007914	[10] From Jax web site: “Importantly, both Ai14 and Ai9 may exhibit low levels of tdTomato expression prior to exposure to Cre recombinase—but the tdTomato expression levels after Cre recombination are greater than those baseline levels. As such, it is recommended that researchers include Cre-negative controls to establish the baseline tdTomato levels in their experiments.”

### Fluorescence imaging

Tissues were imaged in a 35 mm Petri dish using an EVOS FL Auto 2 imaging system with an RFP (531/40 Ex; 593/40 Em) filter cube and a PLAN S-APO 4X, NAO 16, 13 mm objective. COCs, eggs, and blastocysts were imaged in a glass-bottom 35 mm dish in a Zeiss LSM 780 inverted confocal microscope. Photographs of mice were taken with an iPhone using a FS/UL S-02 G2, 525–555 nm wavelength miner’s lamp (Biological Laboratory Equipment).

### Generation of Ikawa *Zp3–Cre* transgenic mouse line

The mouse *Zp3* promoter (JD#445) was a gift from Jurrien Dean (Addgene plasmid #14634; http://n2t.net/addgene:14634; RRID: Addgene_14634). This plasmid contained a 6447 bp fragment of the *Zp3* promoter and a 225 bp bovine growth hormone poly(A) signal. A multicloning site was inserted between the *Zp3* promoter and the poly(A) signal using the following oligonucleotide sequences: forward 5′-ACTAGTCAATTGGATATCCTTAAGCTCGAG-3′ and reverse 5′-CTAGCTCGAGCTTAAGGATATCCAATTGACTAGTGTAC-3′; the final multicloning site was SpeI–MfeI–EcoRV–AflII–XhoI. The full coding sequence of Cre recombinase was then inserted into the SpeI and XhoI sites, removing the intervening restriction sites. The plasmid was linearized with NotI, purified, then microinjected into pronuclei of one-cell C57BL/6 embryos. The embryos were transferred to oviducts of pseudopregnant dams and offspring genotyped for the presence of the Cre recombinase sequence. Positive male offspring were tested for germ line transmission by crossing to transgenic REP08 (Tg (CAG-[floxed neo]-EGFP)08Osb) mice that express EGFP only when the neomycin cassette is excised by Cre recombinase activity [7].

### Generation of *Zp3–iCre* knock-in mice

Cas9 was targeted immediately 3′ of the terminal coding exon of the *Zp3* locus using the following single guide ribonucleic acid (sgRNA) sequence: TTAATGCCTATGTCTGAGATGGG[PAM]. Co-expression of sgRNA, Cas9 nuclease, and puromycin resistance was done with pSpCas9(BB)-2A-Puro (PX459) V2.0 plasmid [9]. The donor plasmid contained (5′ to 3′): 935 bp 5′ homology arm (chr5:136,016,528-136,017,462 mm39), T2A self-cleaving peptide, iCre ORF, minimal SV40 poly(A) signal, and a 431 bp 3′ homology arm (chr5:136,017,518-136,017,948 mm39). Targeting was done in G4 mES cells (B6129F1: 129S6/SvEvTac × C57BL/6Ncr) via co-lipofection of Cas9 delivery plasmid and donor plasmid in a 1:6 molar ratio. Lipofection was done for 8 h, followed by 48 h of puromycin selection (0.9 μg/mL). Following puromycin selection, cells were returned to LIF + serum containing mES media for standard colony formation followed by clonal screening. Clonal screening was done by PCR to amplify across both homology arms and the T2A-iCre-pA genetic payload (*Zp3*–*Cre* Fwd: CCAGTGGTCCAAGCTAGTTTCT, Rev: TGGAGCATGCTAGGAAAGGAAA). Sanger sequencing of PCR amplicons from individual clones was used to confirm mutant allele integrity. Heterozygous clones were intentionally selected for microinjection into albino C57BL/6 J blastocysts for the generation of conventional germline chimeras. Male chimeras were crossed to albino B6 females (JAX: 000058) and their offspring were re-screened to confirm proper transmission of the *Zp3*–*Cre* knock-in allele. Cas9 off-target analysis was not done because the two closest *in silico* predicted potential off-targets were both 4 bp mismatches in non-coding regions.

## Results

**Figure 1:**
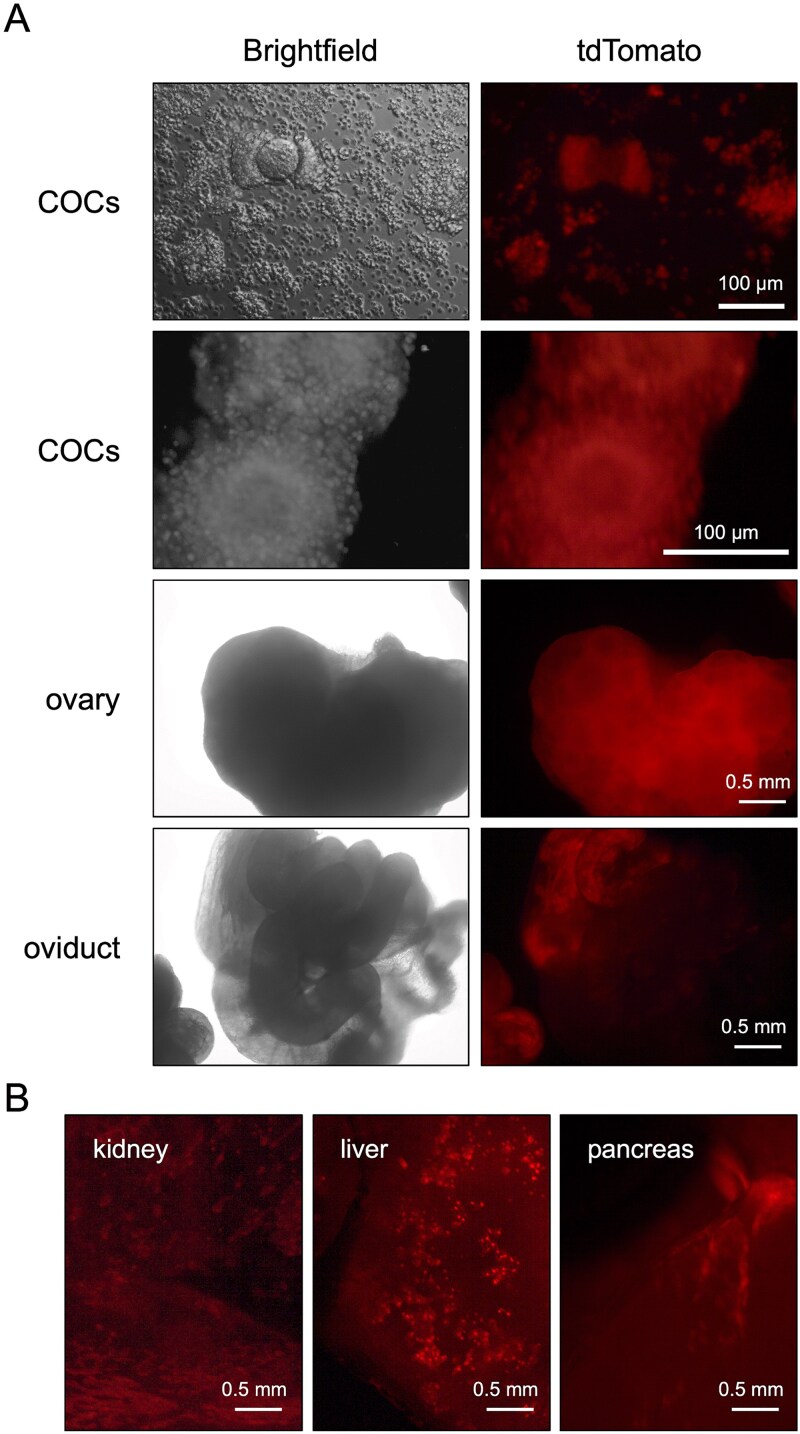
Widespread Cre expression in Marth *Zp3*–*Cre* transgenic mice. tdTomato reporter females were mated to hemizygous Marth *Zp3*–*Cre* males, and different tissues in Cre + adult offspring were dissected and analyzed for tdTomato fluorescence. (A) COCs were isolated from ovaries and imaged as described in Methods. Intact ovaries and oviducts were also imaged. (B) Kidney, liver, and pancreas were collected, and snips of tissue were placed in PBS and imaged. The experiment was performed twice, and representative images are shown.

**Figure 2:**
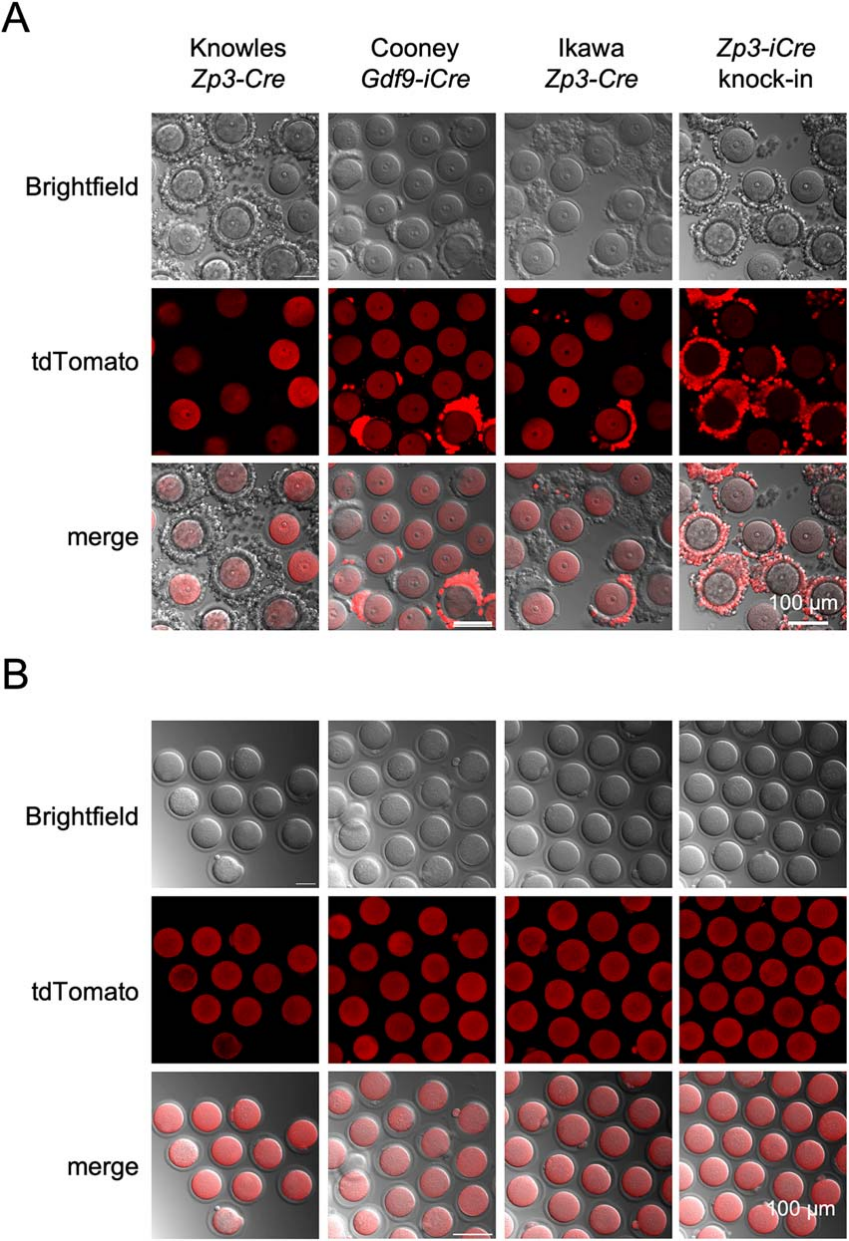
Cre expression in cumulus cells from *Gdf9*–*iCre*, Ikawa *Zp3*–*Cre*, and *Zp3*–*Cre* knock-in models, but not from Knowles *Zp3*–*Cre* mice. tdTomato reporter females were mated to hemizygous males of the genotypes indicated in the figure. Cre + female offspring were superovulated, and GV oocytes (A) and metaphase II eggs (B) were isolated and imaged. The experiment was performed twice, and representative images of brightfield and tdTomato fluorescence are shown.

**Figure 3:**
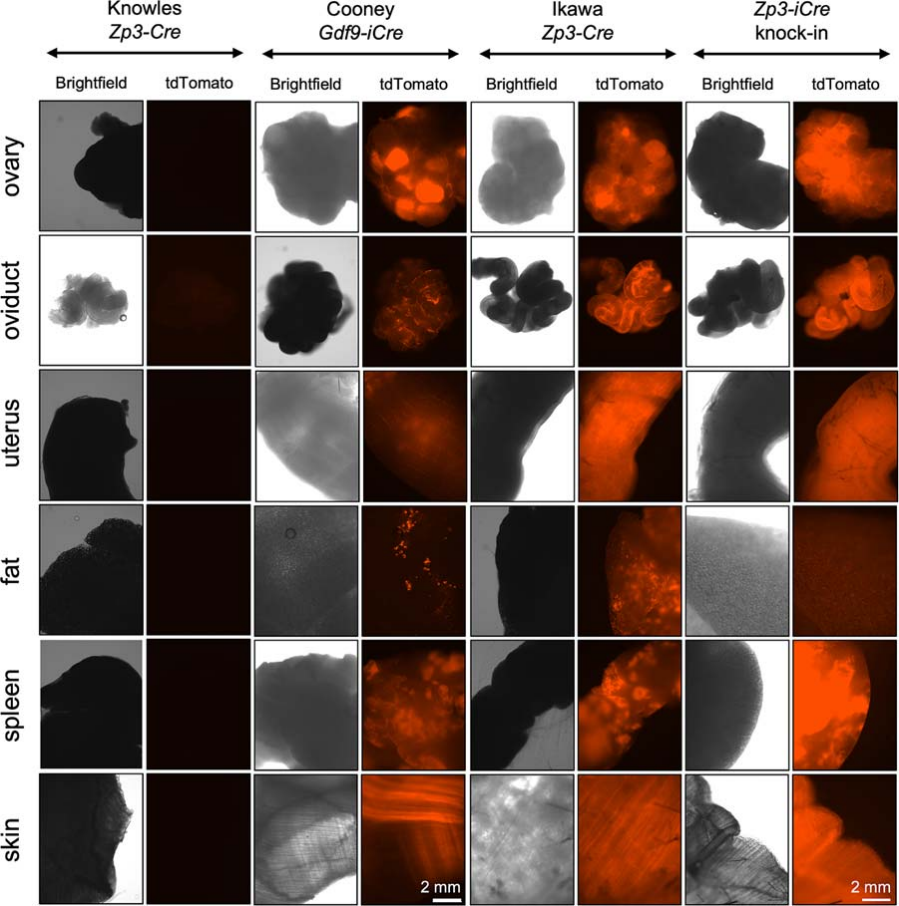
Widespread Cre expression in *Gdf9*–*iCre*, Ikawa *Zp3*–*Cre*, and *Zp3*–*Cre* knock-in models, but not in Knowles *Zp3*–*Cre* mice. tdTomato reporter females were mated to hemizygous males of the genotypes indicated in the figure. Different tissues from adult Cre + female offspring were collected and imaged. The experiment was performed twice, and representative images of brightfield and tdTomato fluorescence are shown.

We began testing for oocyte specificity of Cre expression using the commercially available *Zp3*–*Cre* line developed by Jamey Marth’s group (JAX stock # 006888, Table 1) [2]. Hemizygous *Zp3*–*Cre* males were crossed to female mice homozygous for an allele that results in tdTomato expression only in cells descended from those that had Cre-mediated excision of the floxed region (Ai14 mice) [10]. The Knowles *Zp3*–*Cre* line was tested concurrently. We were quite disappointed to find that offspring from the Marth *Zp3*–*Cre* cross had widespread tdTomato signal in numerous somatic tissues including ovary, oviduct, kidney, liver, and pancreas (Figure 1). As anticipated, the Knowles *Zp3*–*Cre* cross gave offspring with oocyte specific signal; there was strong signal in oocytes and eggs, and no signal in any somatic tissues examined including cumulus cells, other ovarian cell types, oviduct, uterus, fat, spleen, and skin (Figures 2 and 3). Only upon significant overexposure could signal be detected in somatic ovarian tissue (Figure S1).

**Figure 4:**
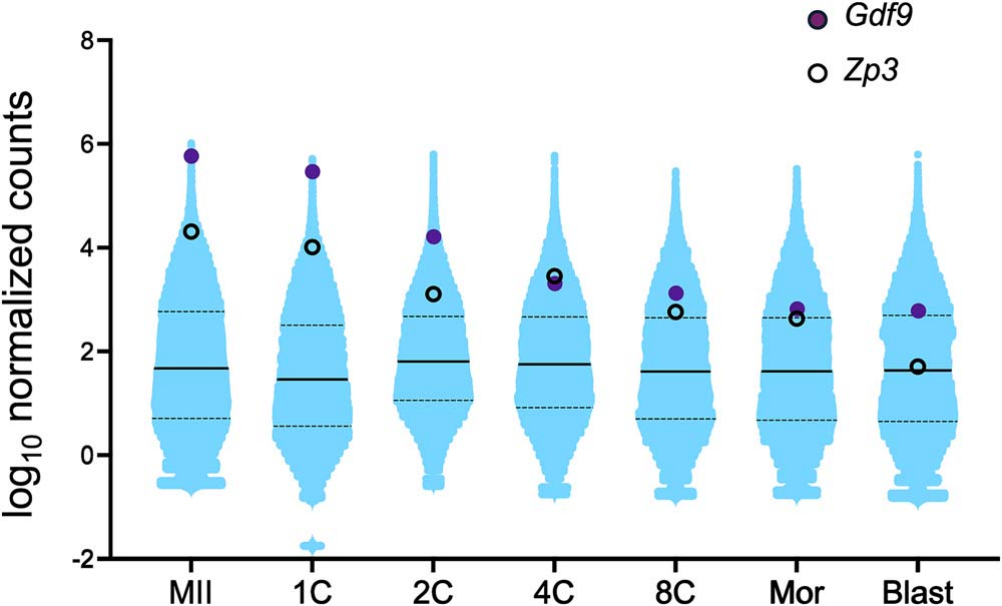
Levels of *Gdf9* and *Zp3* mRNAs are high in early mouse embryos. Violin plots show normalized counts of transcript abundance in eggs and different stages of preimplantation development. Count values from our published RNA-seq data [13] were normalized using DESeq2 [14]. MII: metaphase II eggs, 1C: 1-cell embryo, 2C: 2-cell embryo, 4C: 4-cell embryo, 8C: 8-cell embryo, Mor: morula, Blast: blastocyst.

Another commonly utilized “oocyte-specific” transgenic Cre line is *Gdf9*–*iCre*, which was described as inducing Cre-mediated excision beginning in oocytes within primordial follicles [11]. A more recent report, however, indicates that Cre excision occurs much earlier than that, on embryonic day 13.5 when oocytes enter meiosis [12]. Specificity of Cre expression was tested in [11] by crossing to B6,129-Gtrosa^tm1Sor^ Cre reporter mice, and no β-galactosidase staining was observed in whole mounts of numerous somatic tissues. We crossed hemizygous *Gdf9*–*iCre* males to tdTomato females and examined multiple somatic tissues for red fluorescence. As for the Marth *Zp3*–*Cre* cross, not only oocytes and eggs had tdTomato expression (Figure 2), but widespread tdTomato signal was evident in all somatic tissues examined (Figure 3). This result led us to examine our previous RNA-seq data to determine if *Gdf9* messenger RNA (mRNA) was expressed in preimplantation embryos [13]. Indeed, high expression of *Gdf9* was observed in eggs and all preimplantation embryo stages through blastocyst (Figure 4). This observation provided a possible explanation for the ubiquitous Cre-mediated excision we had observed. Interestingly, *Zp3* mRNA levels were also moderate to high throughout preimplantation development (Figure 4).

Based on these findings, and the published report that the Martin *Zp3*–*Cre* line was not oocyte-specific [1], we decided to generate a new *Zp3*–*Cre* transgenic mouse line using the same promoter region as that used in the original Knowles *Zp3*–*Cre* line. A transgenic construct was made with the ~6.5 kB *Zp3* promoter, Cre recombinase coding region, and a bovine growth hormone poly(A) signal (Figure S2). Transgenic mice were generated using pronuclear microinjection and embryo transfer, resulting in an “Ikawa *Zp3*–*Cre*” founder line that transmitted the transgenic allele. Hemizygous Ikawa *Zp3*–*Cre* males were crossed to tdTomato females and tissue specificity examined. Similar to the Marth *Zp3*–*Cre* line, there was robust signal in oocytes and eggs; however, there was also signal in some cumulus cells (Figure 2), as well as in other ovarian somatic cells, oviduct, uterus, skin, spleen, and fat (Figure 3). To determine if any of the tdTomato signal was a result of leakiness of the tdTomato allele rather than Cre recombinase activity, the signal was checked in Cre-negative littermates. No signal was observed in oocytes or multiple somatic tissues (Figure S3).

**Figure 5:**
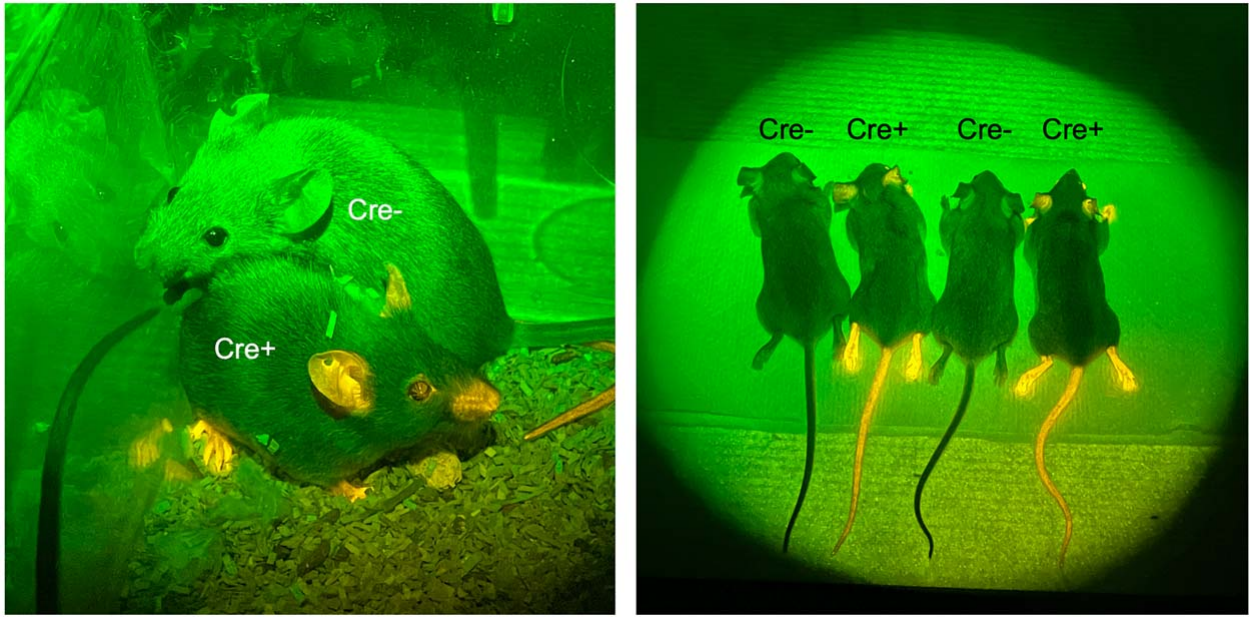
tdTomato fluorescence in skin of offspring from tdTomato x *Zp3*–*Cre* knock-in cross. tdTomato reporter females were mated to hemizygous *Zp3*–*Cre* knock-in males. Digital photos of Cre + and Cre- offspring were taken using an iPhone and a 525–555 nm wavelength filter. Left panel: one Cre- and one Cre + littermate are shown in their cage. Right panel: four euthanized littermates, two Cre- and two Cre + are shown.

Given the difficulties with off-target Cre expression in transgenic *Zp3*–*Cre* and *Gdf9*–*iCre* lines, we decided to generate a mouse line in which Cre recombinase was expressed from the endogenous *Zp3* gene locus. The targeting construct placed Cre at the C-terminal end of the *Zp3* coding region, separated by a T2A self-cleaving peptide [15], and immediately upstream of the stop codon (Figure S4). Standard methodology was used to generate the knock-in allele in XX ES cells and founder mice were generated following ES cell injection into blastocysts. Heterozygous *Zp3*–*Cre* knock-in males were crossed with tdTomato females and the offspring were analyzed. Much to our surprise, offspring carrying the knock-in allele were readily distinguished from their littermates by the floridly pink skin color of the ears, feet, and tails under green light illumination (Figure 5). Examination of oocytes and somatic tissues revealed ubiquitous expression of tdTomato (Figures 2 and 3). This finding would not have been so surprising had the cross included a maternal *Zp3*–*Cre* knock-in allele because there is a significant amount of *Zp3* mRNA detectable in ovulated eggs and preimplantation embryos through the blastocyst stage, likely persistent from maternal stores (Figure 4) [16]. However, the way the cross was done, the *Zp3*–*Cre* knock-in allele was paternal. This finding suggests that *Zp3* is expressed from the paternal allele to some degree during pre- and/or postimplantation embryo development, rather than only in oocytes during the growth phase.

**Figure 6:**
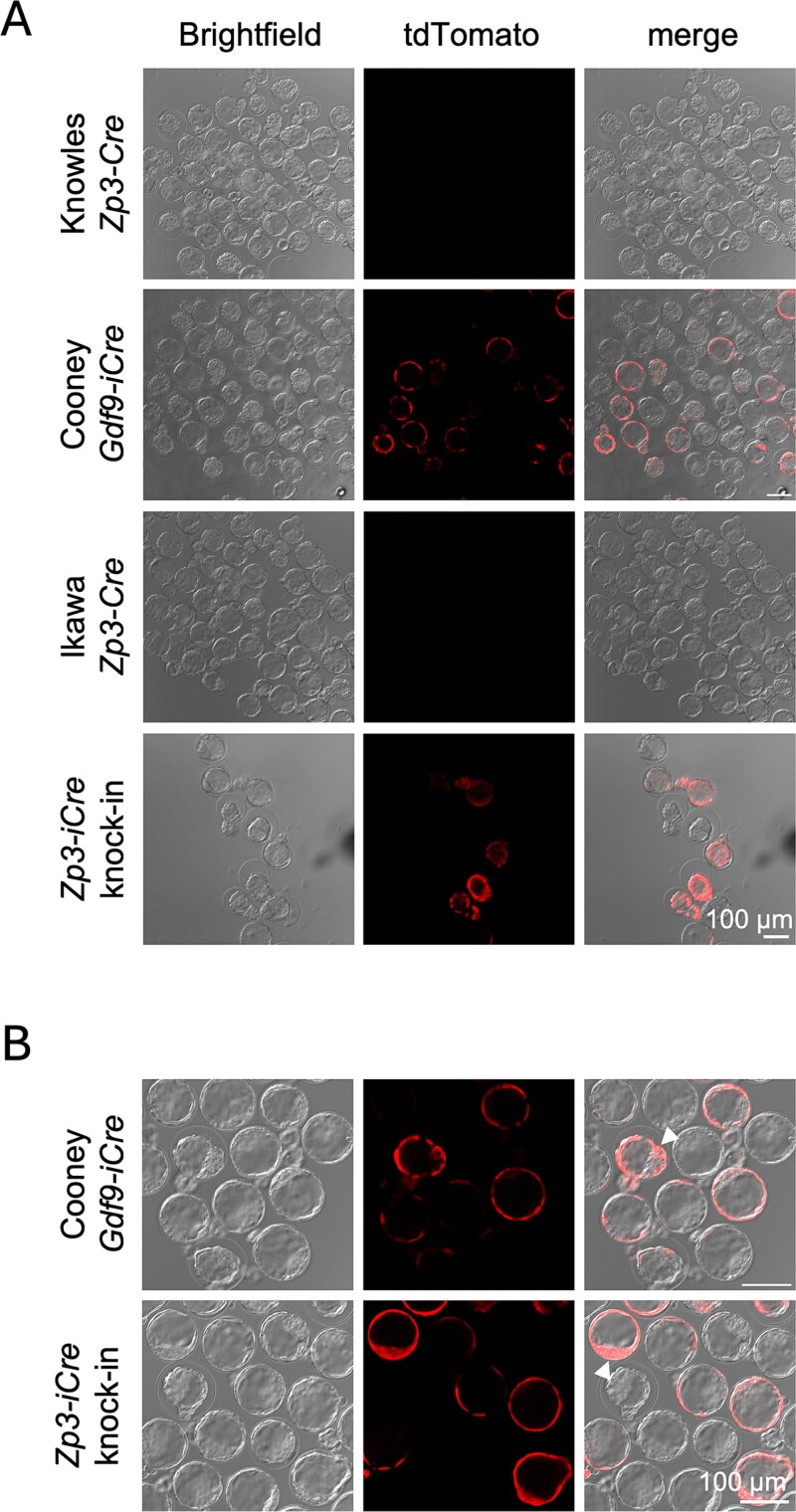
Cre expression in blastocysts from *Gdf9*–*iCre* and *Zp3*–*Cre* knock-in mice. tdTomato reporter females were mated to hemizygous males of the indicated genotypes. Female offspring were superovulated, and 1-cell embryos collected and cultured to the blastocyst stage. The experiment was performed twice, and representative confocal images of brightfield and tdTomato fluorescence in embryos of the different genotypes are shown. The images in (A) were taken with a 10× objective, whereas the images in (B) were taken with a 20× objective. Arrowheads indicate red fluorescence in cells of the inner cell mass.

To determine the timing of Cre recombination across the various *Zp3*–*Cre* lines and the *Gdf9*–*iCre* line, hemizygous *Zp3*–*Cre* or *Gdf9*–*iCre* males were crossed with superovulated tdTomato females and 1-cell embryos were collected and cultured. There was red fluorescence signal by the blastocyst stage in trophectoderm and inner cell mass cells from the *Zp3*–*Cre* knock-in and *Gdf9*–*iCre* lines (Figure 6). No fluorescence signal by the blastocyst stage was observed for the Knowles and the Ikawa *Zp3*–*Cre* transgenic lines. This finding suggests that in the Ikawa *Zp3*–*Cre* line, Cre expression occurred either very late in preimplantation development or in embryonic cells soon after implantation.

## Discussion

Here we performed an extensive series of studies to either identify or generate an alternative mouse oocyte-specific Cre line and failed miserably. Every line tested using the tdTomato mouse, with the exception of the Knowles *Zp3*–*Cre* line, had widespread somatic tissue Cre-mediated excision. Most of the lines clearly expressed Cre from the paternal allele during preimplantation development, resulting in fluorescent signal in blastocyst inner cell mass cells. Such early expression explained the almost ubiquitous Cre-mediated excision in somatic cells of the offspring. Together, these findings led us back to square one—using the original Knowles *Zp3*–*Cre* line for our studies—but recognizing that the extremely high *Mt1* expression in oocytes is an artifact of the transgene [6].

The field has long considered *Zp3* to be an oocyte-specific gene and indeed, *Zp3* is very highly expressed beginning with the early oocyte growth phase [17–19]. However, it is not clear how thoroughly potential *Zp3* transcription in other cell types has been tested. The first report we could find regarding the oocyte specificity of *Zp3* utilized in situ hybridization [19]. The authors stated that *Zp3* expression was oocyte specific because it was not detected in ovarian somatic cells in the context of the whole ovary or in cultured granulosa cells; no other tissues were examined. Subsequent *Zp3* specificity testing was done using Northern blotting and ribonuclease protections assays [18]. *Zp3* mRNA was detectable by these methods in oocytes and eggs, but not in preimplantation embryos through the blastocyst stage or in a wide range of somatic tissues. Importantly, the authors reported that these assays required >1000 copies mRNA/cell for detection.

A recent paper documented that cleavage and blastocyst stage mouse embryos have moderate levels of *Zp3* mRNA based on RNA-seq data [16]; we confirmed this finding (Figure 4). Using immunofluorescence techniques, intracellular ZP3 protein also was documented in preimplantation mouse embryos [16]. These authors concluded that the protein had persisted from the maternal stores rather than being translated during preimplantation embryo development based on an isotopic amino acid labeling assay followed by mass spectrometry. The authors did not speculate on a potential mechanism to explain the lack of mRNA translation in the embryo. Our finding that Cre recombinase was expressed from the paternal *Zp3* gene locus during preimplantation embryo development suggests that at least some of the ZP3 protein present in the preimplantation embryo is newly translated; the isotopic labeling/mass spectrometry assay may not have had the sensitivity to detect this amount. Alternatively, it is possible that the Cre insertion into the *Zp3* locus somehow disrupted post-transcriptional suppression of *Zp3* mRNA translation in the preimplantation embryo, allowing translation of sufficient Cre to excise the floxed allele.

This report is a cautionary tale regarding the importance of documenting the specificity of Cre expression in transgenic or knock-in mouse models using either highly sensitive reporters or by thorough determination of the extent of deletion of the targeted allele. This is particularly important because the efficiency of Cre-mediated deletion depends on the distance between the loxP sites, genomic position, whether or not the floxed allele is heterozygous or homozygous, and even on the age of the mouse [20]. Only after thoroughly characterizing tissue and cell type specificity can an investigator accurately interpret data derived from conditional knockout models. We end with the recommendation that for experiments requiring an oocyte-specific conditional knockout of a gene of interest, the Knowles *Zp3*–*Cre* mouse line appears to be the best choice, while keeping in mind that high *Mt1* expression is likely an artifact of the transgene [6].

## Supplementary Material

Supplemental_File_ioag093

## Data Availability

The data underlying this article will be shared on reasonable request to the corresponding author. Masahito Ikawa holds the position of Associate Editor for Biology of Reproduction and has not peer reviewed or made any editorial decisions for this paper.
