# Supplementary material for: Beware of your “oocyte specific” Cre line: somatic cell Cre expression in several Zp3-Cre lines and the Gdf9-iCre transgenic line
Source: Biol Reprod. Author manuscript; Available in PMC 2026 Jun 7. (PMC13242774; doi:10.1093/biolre/ioag093)
Supplement: supplement [file NIHMS2173270-supplement-supplement.pdf]

Supplemental File for:

Beware of your "oocyte specific" Cre line: somatic cell Cre expression in several *Zp3-Cre* lines and the *Gdf9-iCre* transgenic line

Paula Stein[1], Chihiro Emori[2], Elizabeth Padilla-Banks[1], Lenka Radonova[1], Artiom Gruzdev[3], Masahito Ikawa[2], Carmen J. Williams[1,*]

[1]Reproductive and Developmental Biology Laboratory, National Institute of Environmental Health Sciences, National Institutes of Health, Research Triangle Park, NC, USA.

[2]Research Institute for Microbial Diseases, The University of Osaka, Osaka, Japan.

[3]Gene Editing and Mouse Model Core, National Institute of Environmental Health Sciences, National Institutes of Health, Research Triangle Park, NC, USA.

Contents:

Supplemental Figures 1-4

Fig. S1

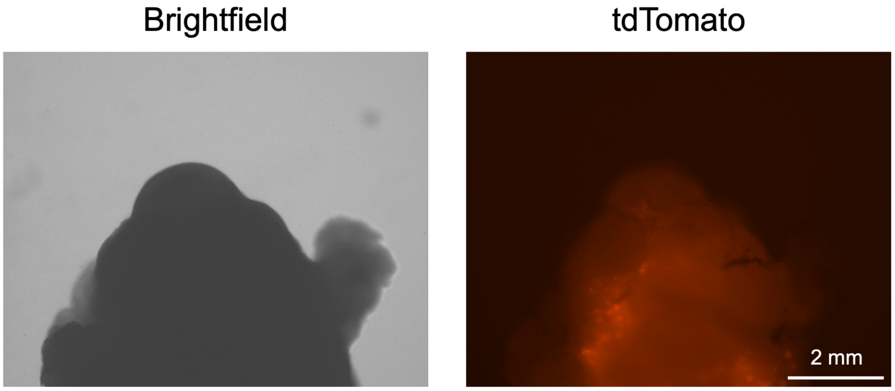

Figure S1. Overexposure is required to detect Cre expression in ovaries of Knowles *Zp3-Cre* line. tdTomato reporter females were mated to hemizygous Knowles *Zp3-Cre* males. Ovaries from adult female offspring were collected and imaged. Utilizing the settings used to image the tissues depicted in Fig. 3, no fluorescence was detected in these ovaries. Only after increasing the exposure to very high values could signal be observed. The experiment was performed twice, and representative images of brightfield and tdTomato fluorescence are shown.

Fig. S2

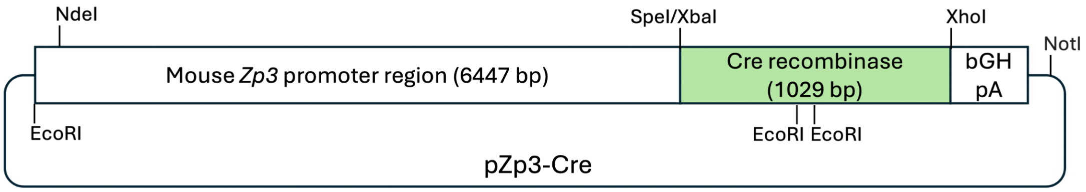

Figure S2. Schematic of the construct used to generate the Ikawa *Zp3-Cre* transgenic mouse line. Restriction enzyme sites are indicated. bGH pA: bovine growth hormone polyadenylation signal.

Fig. S3

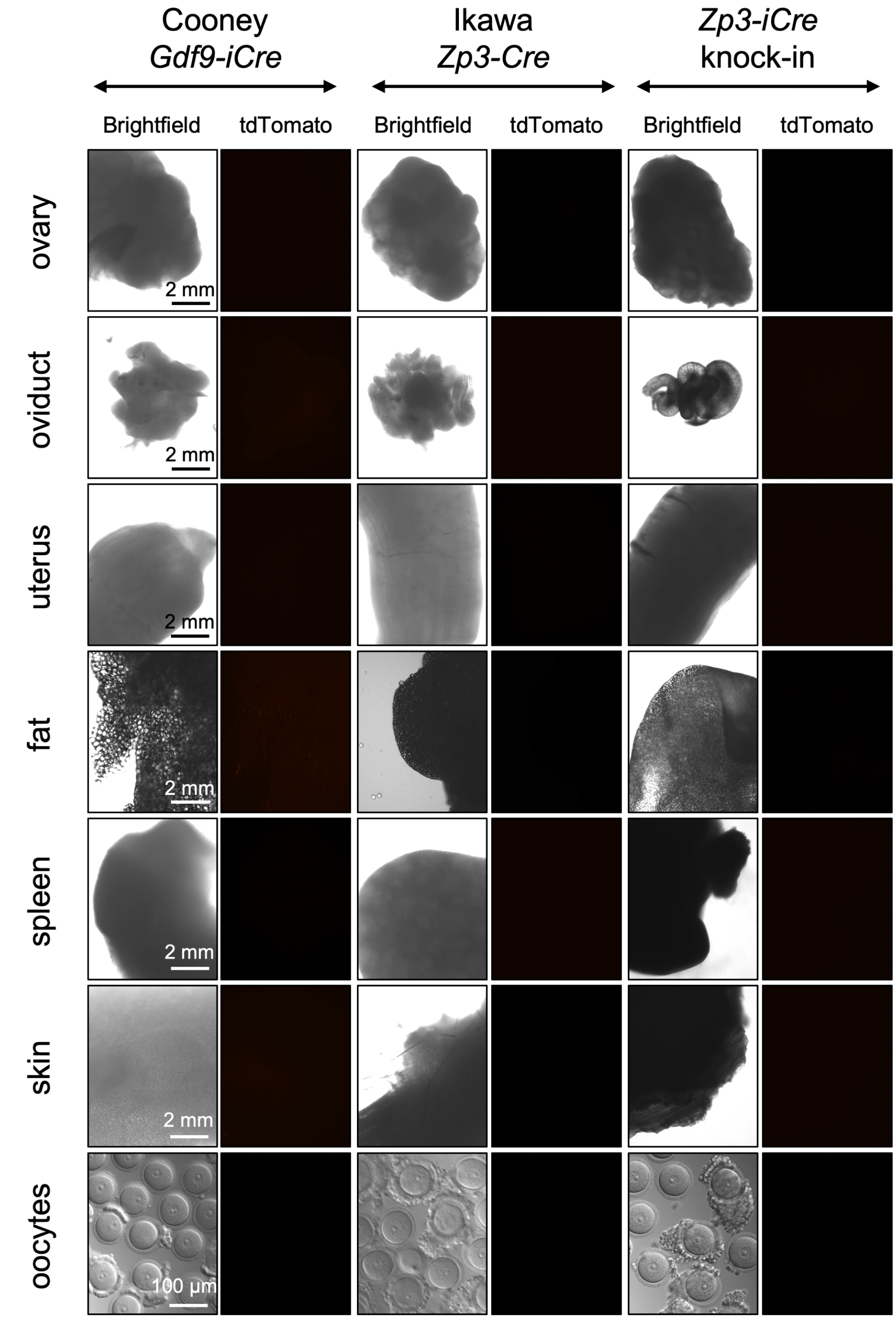

Figure S3. We detect no leakiness from the tdTomato transgene. Cre- negative tdTomato reporter females were mated to hemizygous males of the genotypes indicated in the figure. Different tissues from adult female offspring were collected and imaged. The experiment was performed twice, and representative images of brightfield and tdTomato fluorescence are shown.

Fig. S4

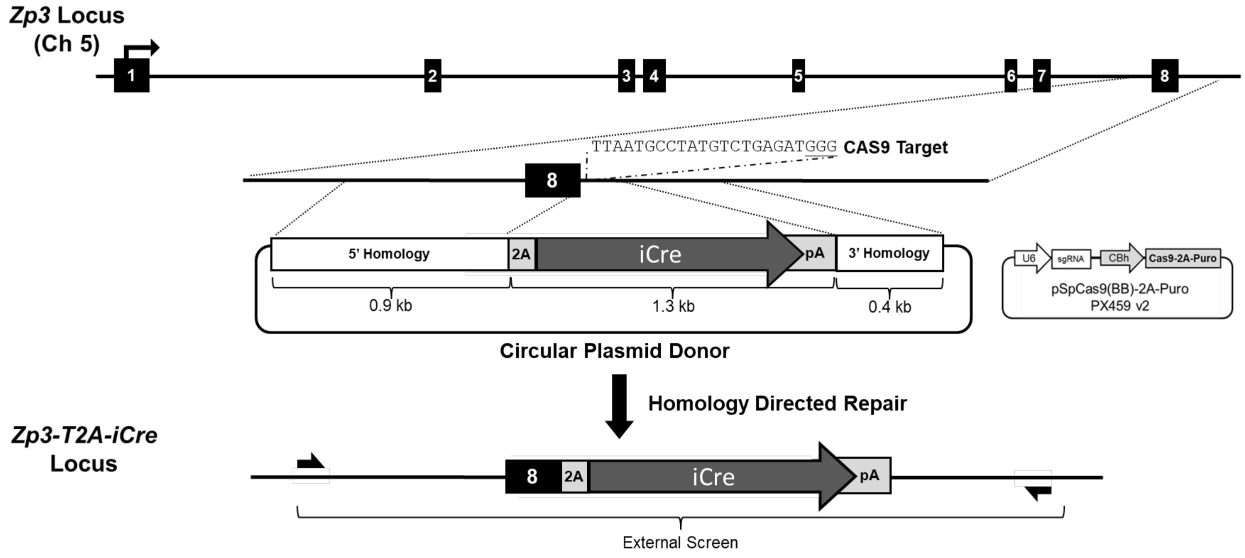

Figure S4. Targeting diagram to generate the Zp3-Cre knock-in mouse line. Mouse ES cells were co-transfected with sgRNA/Cas9/Puromycin resistance expressing plasmid and repair template plasmid. Homology directed repair resulted in the insertion of T2A self-cleaving peptide, mouse codon optimized Cre recombinase ORF (iCre), and minimal SV40 poly(A) signal at the C-terminus coding region of the terminal exon of the Zp3 locus.
